# The Effect of Retinoic Acid on Arsenite-Transformed Malignant UROtsa Bladder Cancer Cells: In Vitro Model of Basal Muscle-Invasive Bladder Cancer

**DOI:** 10.3390/cancers16061178

**Published:** 2024-03-17

**Authors:** Sarmad Al-Marsoummi, Aaron A. Mehus, Scott H. Garrett, Donald A. Sens, Seema Somji

**Affiliations:** Department of Pathology, School of Medicine and Health Sciences, University of North Dakota, Grand Forks, ND 58202, USA

**Keywords:** UROtsa, retinoids, bladder cancer, arsenite

## Abstract

**Simple Summary:**

This study explores the potential use of retinoic acid in treating a severe form of bladder cancer, known as basal muscle-invasive bladder cancer (MIBC), which is among the deadliest cancers in the United States of America. All-trans retinoic acid (tretinoin), commonly used in the differentiation therapy of blood cancer, is investigated in this study for its efficacy against MIBC. By treating bladder cancer cells with tretinoin, the study aimed to determine whether it could diminish the aggressive behavior of these cells. The findings show promising results, suggesting that tretinoin can indeed make the basal muscle cancer cells less aggressive by reducing cell proliferation and increasing differentiation markers. This research could lead to new treatment approaches that improve survival chances for patients with basal muscle-invasive bladder cancer.

**Abstract:**

Bladder cancer (BC) is the eighth most common cause of cancer death in the United States of America. BC is classified into non-muscle-invasive bladder cancer (NMIBC) and muscle-invasive bladder cancer (MIBC). Genetically, MIBCs are categorized into the more aggressive basal subtype or less aggressive luminal subtype. All-trans retinoic acid (tretinoin), the ligand for the RAR-RXR retinoic acid receptor, is clinically used as a differentiation therapy in hematological malignancies. This study aims to determine the effects of retinoic acid on arsenite-transformed malignant urothelial cells (UROtsa As), serving as a model for basal muscle-invasive bladder cancer. We treated three independent isolates of arsenite-transformed malignant human urothelial UROtsa cells (UROtsa As) with tretinoin for 48 h. Cell viability, proliferation, and apoptosis were analyzed using crystal violet staining and flow cytometry. mRNA and protein level analyses were performed using RT-qPCR and the Simple Western™ platform, respectively. Tretinoin was found to reduce cell proliferation and urosphere formation, as well as decrease the expression of basal markers (KRT1, KRT5, KRT6, EGFR) and increase the expression of luminal differentiation markers (GATA3, FOXA1). Mechanistically, the antiproliferative effect of tretinoin was attributed to the downregulation of c-myc. Our results suggest that targeting the retinoic acid pathway can diminish the aggressive behavior of basal muscle-invasive urothelial cancer and may enhance patient survival.

## 1. Introduction

In 2020, an estimated 573,278 individuals worldwide were diagnosed with bladder cancer. In 2023, an estimated 82,290 adults in the United States of America were diagnosed with bladder cancer [1]. Bladder cancers can be histologically categorized as muscle-invasive or non-muscle-invasive. Molecular profile analysis categorizes muscle-invasive bladder cancers into a less aggressive luminal subtype and a more aggressive basal subtype [2,3]. While both luminal and basal subtypes are characterized by muscle invasion, they differ in survival rates and require different therapeutic approaches [2,4,5].

Arsenite (As+3) is an environmental toxicant that has been linked to the development and progression of bladder cancer. Regions with elevated levels of arsenite in their drinking water have been found to exhibit a higher risk and prevalence of bladder cancer [6,7,8]. Our laboratory has previously induced malignant transformation in immortalized non-tumorigenic urothelial cells (UROtsa cells) through prolonged exposure to arsenite and established multiple independent malignant UROtsa cell isolates [9,10]. Malignant UROtsa cells transformed with arsenite demonstrate tumorigenicity in athymic mice with xenografts that mimic basal muscle-invasive bladder cancer with high expression of the basal markers KRT5, KRT6, KRT1, and EGFR [11].

Retinoids are compounds derived from vitamin A that bind to and activate nuclear retinoic acid receptors (RARs) and retinoid X receptors (RXRs) [12]. Retinoids have long been of interest to researchers due to their roles in cell differentiation, growth, and apoptosis [12]. The clinical success of retinoids in inducing differentiation and complete remission in patients with acute promyelocytic leukemia [13,14] has encouraged investigation of the potential use of retinoids in solid, non-hematological malignancies [15], including bladder cancer [16]. Meta-analysis studies have shown an association between low blood vitamin A levels and low dietary vitamin A intake and a higher risk of bladder cancer development [17]. Retinoic acid (all-trans retinoic acid (ATRA), tretinoin) is an active metabolite of vitamin A that can modify both the retinoic acid receptor (RAR) and the retinoid X receptor (RXR) [18]. In vitro studies have shown variable effects of retinoids on bladder cancer cell lines (T24, RT4, 5637, RT112, and HT-1376 cells), ranging from a cytostatic effect, stimulation of proliferation, and inhibition of EGF-stimulated cell proliferation to direct activation of PPARγ [19,20,21,22]. In vivo studies have been limited, primarily focusing on the effect of dietary vitamin A in preventing bladder cancer development [16], with scarce data available on the effect of retinoids on bladder cancer xenografts in animal models. Limited data are available regarding the effect of retinoids on the distinct molecular subtypes of bladder cancer, particularly the effect of retinoids on basal muscle-invasive bladder cancer.

We hypothesized that tretinoin could reduce the aggressiveness of arsenite-transformed malignant UROtsa cells in a basal muscle-invasive bladder cancer cell model and induce a favorable molecular expression profile. We treated different isolates of malignant UROtsa cells with different doses of tretinoin and investigated the effect on cell viability, proliferation, and the expression of key basal and luminal markers. Our data showed that tretinoin reduced the aggressiveness of malignant UROtsa cells by decreasing cell proliferation and the formation of urospheres, downregulating the expression of basal markers (KRT5, KRT6, KRT1, EGFR), and upregulating the expression of luminal markers (GATA3 and FOXA1).

Our findings establish a potential differentiation effect of retinoids in basal muscle-invasive bladder cancer cases with poor prognosis and provide an avenue for potential progress in precision medicine and the use of retinoids in the treatment of muscle-invasive bladder cancer.

## 2. Materials and Methods

### 2.1. Cell Culture

Arsenite-transformed UROtsa cells (UROtsa As_1, UROtsa As_2, UROtsa As_3) were previously established and characterized in our laboratory [9,23,24]. Cells were cultured at 37 °C and 5% CO_2_ in Dulbecco’s modified Eagle’s medium (DMEM) (ThermoFisher Scientific, Waltham, MA, USA, Catalog #31600-034) supplemented with 5% fetal bovine serum (FBS). Tretinoin (retinoic acid) was purchased from Selleck Chemicals (Houston, TX, USA, Catalog #S1653).

### 2.2. Viral Vector

Cells were seeded into a 12-well plate at 20% confluency and allowed to adhere for 24 h. Cells were transduced with 8000 viral particles per cell with an adenoviral vector expressing C-MYC (AdC-MYC), purchased from Vector Biolabs (Malvern, PA, USA, #1285), in 1 mL of culture medium. After 24 h, the culture medium was replaced with fresh culture medium containing tretinoin, and the cells were allowed to grow for an additional 72 h.

### 2.3. Cell Viability and Clone Formation Assay

Cells were seeded into a 12-well plate at 20% confluency and allowed to adhere for 24 h. The culture medium was then refreshed with one that contained retinoic acid, and cells were allowed to grow for 48–72 h according to the experimental protocol at 37 °C and 5% CO_2_. For the clone formation assay, cells were seeded at a density of 500 cells per cm^2^ in a six-well plate and allowed to grow for 10 days.

For crystal violet staining, the medium was discarded, and cells were quickly washed with deionized, double-distilled water (DDH2O). Cells were then incubated with 0.5% crystal violet containing 20% methanol for 15 min, after which the cells were washed with DDH2O and allowed to air-dry overnight. Cells were lysed using 0.1 M sodium citrate in 25% ethanol (pH 4.0), and the absorbance at a wavelength of 595 nm was measured using a microplate spectrophotometer (BioTek EL800, Santa Clara, CA, USA).

### 2.4. Flow Cytometry Analysis

#### 2.4.1. Proliferation Study

Cells were incubated with 5 µM of Tag-it Violet™ dye (BioLegend, San Diego, CA, USA, #425101) in phosphate-buffered saline (PBS) at 37 °C for 30 min, followed by quenching of the Tag-it Violet™ stain by adding five times the original volume of culture medium, which was then followed by centrifugation at 350× *g*. The cells were seeded at 20% confluency. After 24 h, the cells were treated with a fresh culture medium containing tretinoin or DMSO and allowed to grow for 48 h. Cells were detached using the TrypLE enzyme (Thermo Fisher Scientific, Waltham, MA, USA, #12563029), collected in Falcon™ tubes, and centrifuged at 350× *g*, followed by resuspension in FACS buffer (PBS and 5% FBS).

#### 2.4.2. Apoptosis Study

Cells were cultured in culture medium supplemented with DMSO or 5, 10, or 20 µM of tretinoin for 48 h. Cells were detached using the TrypLE enzyme, and supernatants were collected. The cells were then stained for both annexin V and propidium iodide (PI) using a Pacific Blue Annexin V Apoptosis Kit (BioLegend, #640928) according to the manufacturer’s protocol and analyzed through flow cytometry (Sony SH800).

Flow cytometry data were analyzed using FlowJo software v10.10.0 (BD Life Sciences, Franklin Lakes, NJ, USA).

### 2.5. RNA Extraction and RT-qPCR

Cells were lysed with 350 μL of RLT^®^ buffer, which has a proprietary composition (Qiagen, Hilden, Germany). Qiashredder tubes (Qiagen, #79656) were used to dissociate cells according to the manufacturer’s protocol. The RNeasy Mini Plus Kit and the QIAcube instrument, purchased from Qiagen (#74636), were used to extract RNA using the manufacturer’s protocol. Purified RNA was quantified using a NanoDrop spectrophotometer (Thermo Fisher Scientific). A total of 1 µg of cDNA was synthesized from the total purified RNA using the LunaScript^®^ RT SuperMix Kit (New England Biolabs, Ipswich, MA, USA, #E3010L) according to the manufacturer’s protocol. cDNA was diluted with nuclease-free water to achieve a final concentration of 10 ng/µL.

A total of 20 ng of cDNA was used in a 20 µL qPCR reaction and analyzed using the BioRad CFX96 Touch Real-Time PCR thermocycler (Hercules, CA, USA) and the Luna^®^ Universal qPCR Master Mix (New England Biolabs, #M3003E). qPCR thermocycler conditions were one cycle of 2 min at 95 °C, forty cycles of 5 s at 95 °C, and 30 s at the annealing temperature of 60 °C. Expression levels were determined using threshold cycle values (Cq) and the 2^−∆∆Ct^ method. RPLP0 or ACTB were used as reference genes. Primers were purchased from IDT (Coralville, IA, USA); the primers used are listed in the Supplementary Materials (Table S1).

### 2.6. Urosphere Formation Assay

Cells were cultured in a T-25 flask and allowed to reach confluency, after which they were washed three times with PBS, detached with the TrypLE enzyme before then being centrifuged at 450× *g*, and resuspended in a serum-free spheroid culture medium composed of a 1:1 mixture of Dulbecco’s modified Eagle’s medium (DMEM) and Ham’s F-12 growth medium supplemented with selenium (5 ng/mL), insulin (5 μg/mL), transferrin (5 μg/mL), hydrocortisone (36 ng/mL), triiodothyronine (4 pg/mL), and epidermal growth factor (10 ng/mL). Cells were strained using a 40-micron cell strainer and seeded into a Corning™ 6-well ultra-low attachment plate (Corning, Corning, NY, USA, #3471) at a density of 1000 cells/cm^2^. Each well received 3 mL of the serum-free spheroid culture medium supplemented with either DMSO as a control or tretinoin. Cells were allowed to grow undisturbed for ten days at 37 °C in a 5% CO_2_ incubator. The spheres were harvested from the well and transferred to a 15 mL tube. The well was rinsed with 2 mL of DMEM and combined with the collected suspension before then being centrifuged at 200× *g* for five minutes. The supernatant was carefully aspirated, ensuring the sphere pellet was not disturbed, followed by gentle resuspension to a final volume of 500 µL with DMEM. Fifty microliters of the sphere suspension was added to each well of the 96-well plate, and the spheres were visualized and counted in each well under the microscope.

### 2.7. Protein Analysis by ProteinSimple

Simple Western™ blotting was used to quantify and analyze protein expression as previously described [25]. Cells were washed twice with ice-chilled phosphate-buffered saline and lysed in radio-immunoprecipitation assay (RIPA) lysis buffer supplemented with PMSF, a protease inhibitor cocktail, and sodium orthovanadate (Santa Cruz Biotechnology, Dallas, TX, USA). The cell lysate was sonicated twice for 15 s each time and centrifuged at 12,000× *g* for 15 min. Protein concentration was determined using the Pierce bicinchoninic acid (BCA) protein assay kit (Thermo-Scientific Pierce, Waltham, MA, USA). Protein lysates were combined with 5× fluorescent master mix (ProteinSimple, San Jose, CA, USA) containing dithiothreitol, fluorescent standards, and a system control protein (26 kDa), which was followed by denaturation of the protein lysate by heating (95 °C for 5 min) to denature the protein lysate. Protein lysate was separated, and immunodetection of target proteins was performed using a capillary-based Jess Simple Western™ instrument (ProteinSimple, San Jose, CA, USA) according to the manufacturer’s protocol. The ProteinSimple system control protein (26 kDa) was used as an internal control to normalize protein expression. The concentrations of protein lysate and the antibodies/dilutions used are listed in the Supplementary Materials (Table S2). The uncropped blots are also provided in the Supplementary Materials.

### 2.8. Statistical Methods and In Silico Platforms

Data were expressed as means ± SEM and analyzed using GraphPad Prism v10 via one-way ANOVA with appropriate post-hoc testing. All experiments were repeated at least three times. For survival analysis, Kaplan–Meier analysis (log-rank *p*-value) was used to analyze the correlation between gene expression level and survival probability using the Human Protein Atlas (www.proteinatlas.org, accessed on 5 January 2024) [26] and GEPIA2 (http://gepia2.cancer-pku.cn/#survival, accessed on 8 March 2024) [27] datasets. In our analysis, we used both optimal expression cut-off values and median expression values. The optimal expression cut-off is defined as the FPKM (fragments per kilobase of transcript per million mapped reads) value that maximizes the survival difference between two groups while achieving the lowest log-rank *p*-value. This cut-off was determined through survival analysis. The median expression, on the other hand, refers to the median FPKM value computed from the gene expression data of all patients included in this dataset.

## 3. Results

### 3.1. Tretinoin Reduces Cell Proliferation in Malignant UROtsa Cells

**Figure 1:**
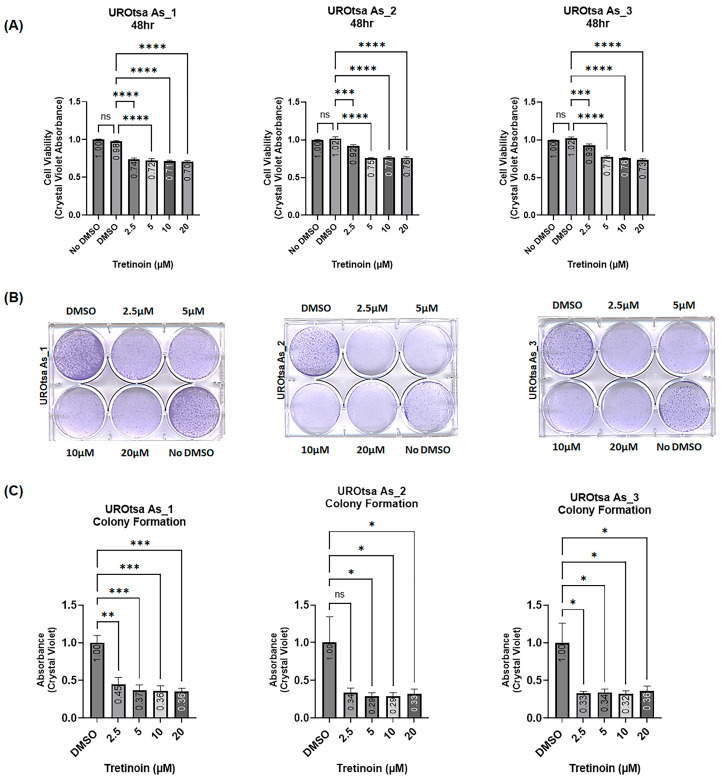
Tretinoin reduces viable cell numbers and colony formation of the malignant UROtsa cells. (**A**) Cell viability analyzed by crystal violet staining, illustrating the effect of 48 h treatment with 2.5, 5, 10, and 20 µM of tretinoin on three independent isolates of malignant arsenite-transformed UROtsa cells (UROtsa As_1, UROtsa As_2, UROtsa As_3). (**B**,**C**) Colony formation assay with representative micrographs illustrating the effect of 10 days of treatment with 2.5, 5, 10, and 20 µM of tretinoin on the colony formation potential of UROtsa As_1, UROtsa As_2, and UROtsa As_3 cells. Treatment with 2.5, 5, 10, and 20 µM of tretinoin significantly reduced both cell numbers and colony-forming capacity in three independent cell isolates of malignant arsenite-transformed UROtsa cells (*n* = 3 for each cell isolate, one-way ANOVA; ns = not significant; * *p* < 0.05, ** *p* < 0.01, *** *p* < 0.001, **** *p* < 0.0001).

This laboratory previously transformed immortalized non-malignant human urothelial cells (UROtsa) into malignant cells (UROtsa As) through prolonged exposure to arsenite [9]. We established multiple independent cell isolates, all of which were tumorigenic in immunodeficient mice, and modeled the basal muscle-invasive bladder cancer [23,24]. Treating three independent cell isolates of arsenite-transformed malignant UROtsa cells (UROtsa As_1, UROtsa As_2, and UROtsa As_3) with 2.5, 5, 10, and 20 µM of tretinoin (retinoic acid) for 48 h significantly reduced the number of viable cells in all three isolates, as measured by crystal violet staining (Figure 1A). The tretinoin dose–time response is available in the Supplementary Materials (Figure S1). Furthermore, tretinoin treatment for ten days significantly reduced the number of colonies formed in all three cell isolates (Figure 1B,C). The antiproliferative effect of tretinoin was consistently significant across all three cell isolates, with a clear dose-dependent response observed in UROtsa As_1 and UROtsa As_3 cells (Figure 1A), though no dose-response effect was observed in the colony formation assay (Figure 1B,C).

### 3.2. Tretinoin Reduces Cell Proliferation with Minimal Apoptosis in Malignant UROtsa Cells

**Figure 2:**
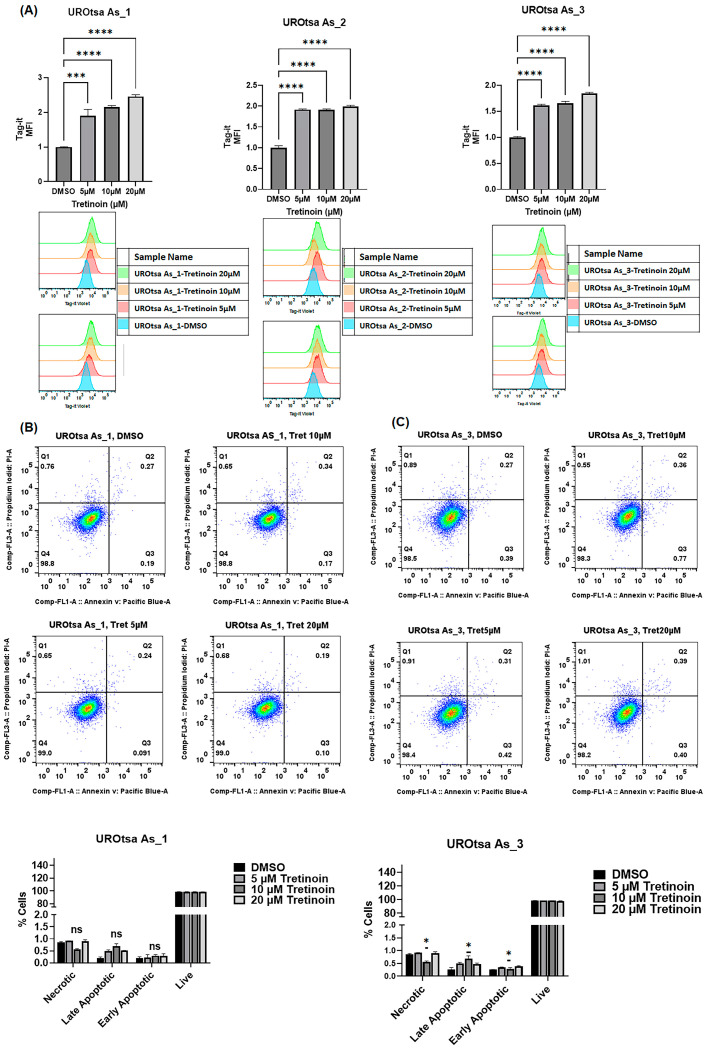
Tretinoin reduces cell proliferation in malignant UROtsa cells. (**A**) Flow cytometry analysis of cell proliferation using Tag-it Violet™ cell tracking dye. Treatment with 5, 10, and 20 µM of tretinoin significantly reduced cell proliferation, as indicated by the increased retention (mean fluorescence intensity (MFI)) of Tag-it Violet™ dye in UROtsa As_1, UROtsa As_2, and UROtsa As_3 cells. (**B**,**C**) Apoptosis assay by flow cytometry analysis of annexin V and propidium iodide in UROtsa As_1 and UROtsa As_3 cells treated with 5, 10, and 20 µM of tretinoin for 48 h demonstrates that 10 µM of tretinoin minimally but significantly induced apoptosis in UROtsa As_3 cells (*n* = 3, one-way ANOVA; ns = non-significant; * *p* < 0.05; *** *p* < 0.001; **** *p* < 0.0001). Data represented as mean ± SEM.

To confirm that the reduction in cell numbers induced by tretinoin in malignant UROtsa cells represents an actual reduction in cell proliferation, we used Tag-it Violet™ cell proliferation and tracking dye. Our results confirmed a dose-dependent decrease (illustrated by increased retention of Tag-it dye) in cell proliferation in UROtsa As_1 and UROtsa As_3 after treatment with 5, 10, and 20 µM of tretinoin (Figure 2A) for 48 h, with UROtsa As_2 showing a similar antiproliferative response after treatment with 5 µM and 10 µM of tretinoin.

Apoptosis analysis using annexin V and propidium iodide in flow cytometry of UROtsa As_1 and UROtsa As_3 cells showed that treatment with 10 µM of tretinoin for 48 h marginally but significantly increased apoptotic cell death by 0.5% in only UROtsa As_3 (Figure 2B), with no significant apoptotic cell death observed at a dose of 5 µM or 20 µM, or in UROtsa As_1 (Figure 2A). UROtsa As_2 showed a comparable apoptotic response after tretinoin treatment (Figure S2).

### 3.3. Tretinoin Reduces the Urosphere Formation Capacity of Malignant UROtsa Cells

**Figure 3:**
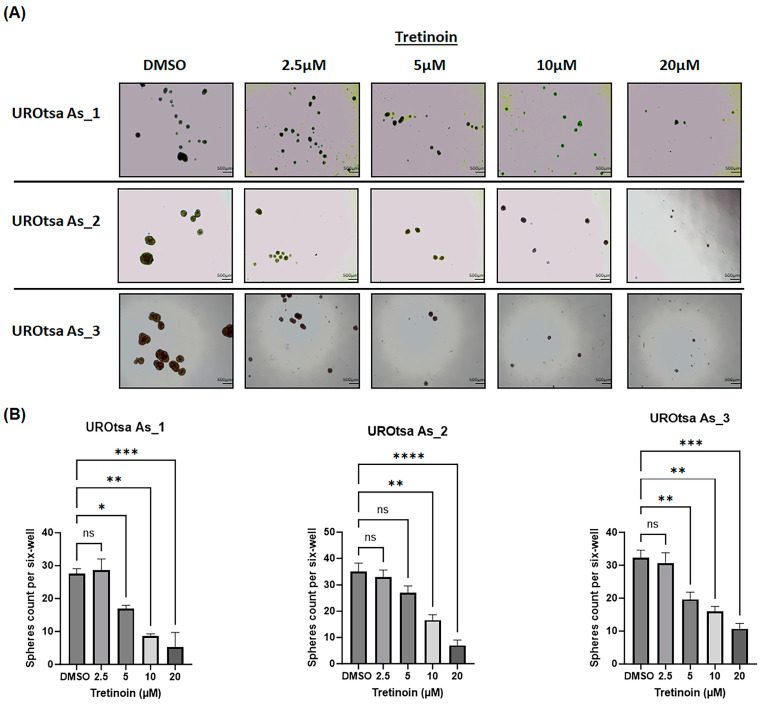
Tretinoin reduces urosphere formation in malignant UROtsa cells. (**A**) Representative microscopy images of urosphere formation in UROtsa As_1, UROtsa As_2, and UROtsa As_3 cells (scale bar = 500 µm). (**B**) Urosphere formation assay that demonstrates the effect of tretinoin on the number of urospheres formed in UROtsa As_1, UROtsa As_2, and UROtsa As_3 cells. Cells were grown on ultra-low attachment plates in serum-free medium for ten days and treated with either DMSO (as a control) or 2.5, 5, 10, or 20 µM of tretinoin. Images were captured at 100× magnification (*n* = 3, one-way ANOVA; * *p* < 0.05, ** *p* < 0.01, *** *p* < 0.001, and **** *p* < 0.0001). Data represented as mean ± SEM.

Arsenite-transformed malignant UROtsa cells can form urospheres when grown in serum-free medium under low attachment conditions [10,23], indicating the self-renewal and stem cell characteristics of these cells. Treatment of UROtsa As_1, UROtsa As_2, and UROtsa As_3 with 5, 10, or 20 µM of tretinoin caused a dose-dependent decrease in the number of urospheres formed in all three cell isolates (Figure 3), with no significant reduction observed with 2.5 µM of tretinoin.

### 3.4. Tretinoin Reduces the Expression of Basal Markers in Malignant UROtsa Cells

**Figure 4:**
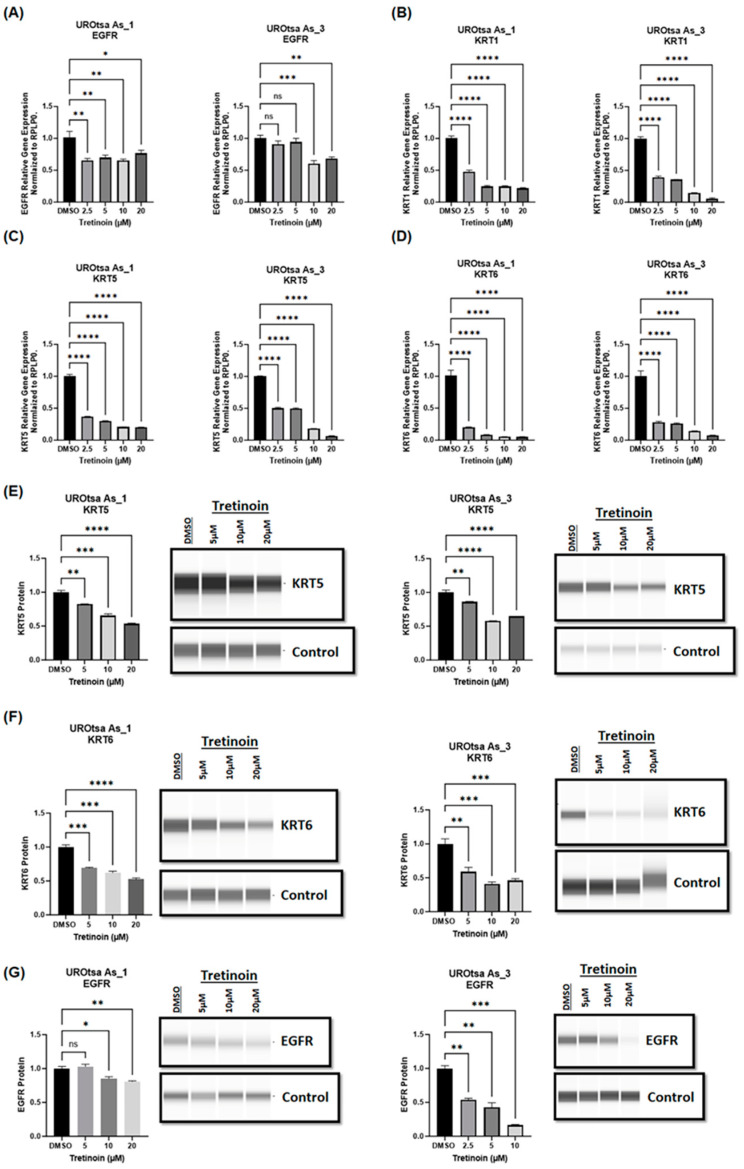
Tretinoin reduces the expression of basal markers in malignant UROtsa cells. mRNA levels were analyzed through RT-qPCR of (**A**) EGFR, (**B**) KRT1, (**C**) KRT5, and (**D**) KRT6 in UROtsa As_1 and UROtsa As_3 cells after treatment with either DMSO (as a control) or 2.5, 5, 10, or 20 µM of tretinoin for 48 h. Protein levels were analyzed through Simple Western™ blotting of (**E**) KRT5, (**F**) KRT6, and (**G**) EGFR proteins in UROtsa As_1 and UROtsa As_3 cells after treatment with 5, 10, or 20 µM of tretinoin for 48 h (*n* = 3, one-way ANOVA; ns = non-significant; * *p* < 0.05, ** *p* < 0.01, *** *p* < 0.001, **** *p* < 0.0001). Data represented as mean ± SEM.

Our laboratory demonstrated that arsenite-transformed malignant UROtsa cells exhibit characteristics of the basal muscle-invasive phenotype of bladder cancer, including high expression of basal markers such as EGFR, KRT1, KRT5, and KRT6 [10,23,24]. Therefore, we sought to determine whether tretinoin, as a differentiation drug, could change the basal gene expression profile of malignant UROtsa cells. Treatment with tretinoin (2.5, 5, 10, 20 µM) for 48 h significantly reduced the mRNA levels of the basal markers *EGFR* (Figure 4A), *KRT1* (Figure 4B), *KRT5* (Figure 4C), and *KRT6* (Figure 4D) in both UROtsa As_1 and UROtsa As_3 cells. Furthermore, the analysis of protein levels using Simple Western™ blotting showed a comparable significant reduction in the protein levels of KRT5 (Figure 4E), KRT6 (Figure 4F), and EGFR (Figure 4G) in both UROtsa As_1 and UROtsa As_3 cells. Similar results were observed for UROtsa As_2 cells (Supplementary Materials Figure S5).

### 3.5. Tretinoin Increases the Expression of Luminal Markers in Malignant UROtsa Cells

**Figure 5:**
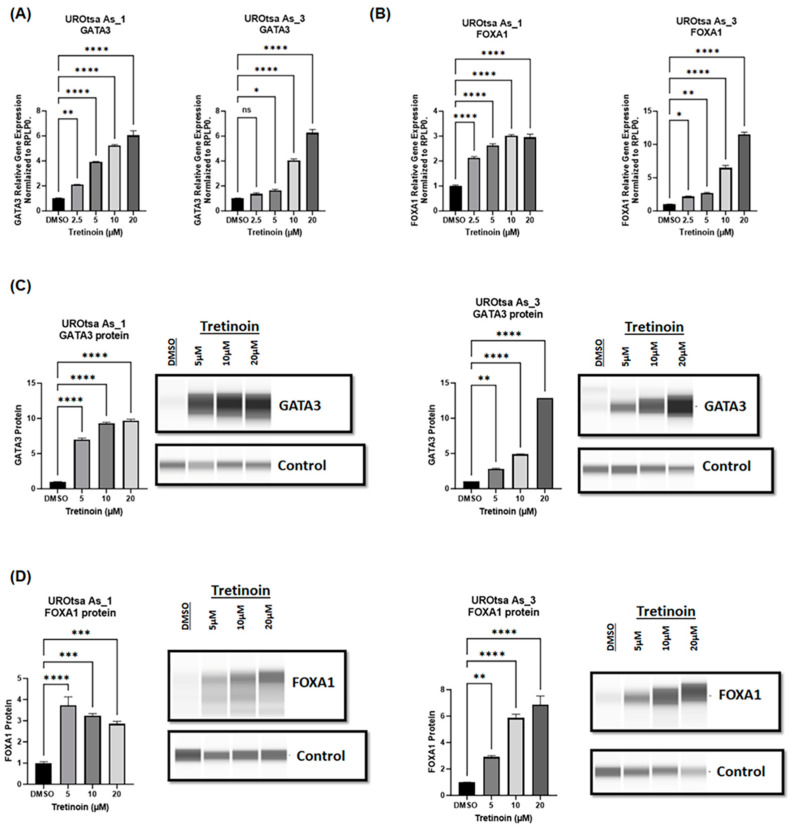
Tretinoin increases the expression of luminal markers in malignant UROtsa cells. mRNA levels were measured through RT-qPCR of (**A**) *GATA3* and (**B**) *FOXA1* in UROtsa As_1 and UROtsa As_3 cells after treatment with 2.5, 5, 10, or 20 µM of tretinoin for 48 h. Protein levels were analyzed through Simple Western™ blotting of (**C**) GATA3 and (**D**) FOXA1 in UROtsaAs_1 and UROtsa As_3 after treatment with 5, 10, or 20 µM of tretinoin for 48 h (*n* = 3, one-way ANOVA; ns = non-significant; * *p* < 0.05, ** *p* < 0.01, *** *p* < 0.001, **** *p* < 0.0001). Data represented as mean ± SEM.

Malignant arsenite-transformed UROtsa cells mimic basal muscle-invasive bladder cancer and are characterized by low expression of luminal markers (GATA3, FOXA1) [24]. Since tretinoin is established as a differentiation therapy in acute leukemia [11], we sought to determine whether tretinoin treatment could induce the expression of GATA3 and FOXA1, which are key markers driving the luminal phenotype of bladder cancer [28,29]. A 48 h treatment with tretinoin induced a significant, dose-dependent increase in the mRNA levels of *GATA3* (Figure 5A) and *FOXA1* (Figure 5B) in both UROtsa As_1 and UROtsa As_3 cells. Protein analysis through Simple Western™ blotting showed a comparable dose-dependent increase in GATA3 and FOXA1 protein levels (Figure 5C,D) in both UROtsa As_1 and UROtsa As_3 cells after treatment with 5, 10, or 20 µM of tretinoin for 48 h. Similar results were observed for UROtsa As_2 cells (Supplementary Materials Figure S5).

### 3.6. Tretinoin-Induced Changes in Gene Expression Correlate with Better Survival in Bladder Cancer Patients

**Figure 6:**
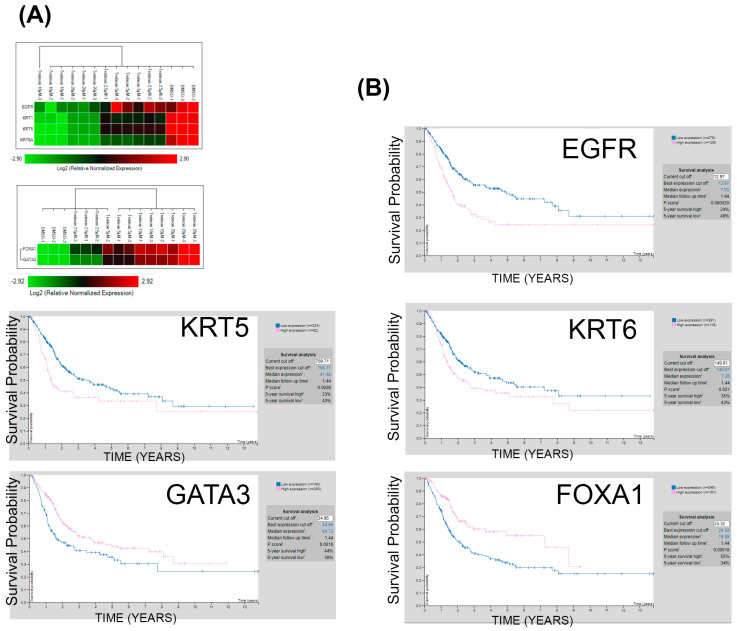
Tretinoin induces molecular expression profiles favoring survival in bladder cancer. (**A**) Heatmap showing the effect of 48 h of tretinoin treatment on *EGFR*, *KRT5*, *KRT6*, *GATA3*, and *FOXA1* gene expression in malignant UROtsa cells. (**B**) Kaplan−Meier survival analysis indicates that low expression of *EGFR*, *KRT5*, and *KRT6* is correlated with poor survival. In contrast, high expression of *FOXA1* and *GATA3* is associated with better survival in patients with bladder cancer.

To investigate the prognostic significance of tretinoin-induced gene expression changes in malignant UROtsa cells (Figure 6A), which serve as a bladder cancer cell model, we analyzed the association between the expression levels of these specific genes and survival probability in bladder cancer patients. This analysis utilized the publicly available Human Protein Atlas dataset [26]. Interestingly, lower expressions of *EGFR*, *KRT5*, and *KRT6* are associated with better survival in bladder cancer patients (Figure 6B). Similarly, higher expression of *FOXA1* or *GATA3* is linked to improved survival (Figure 6B). The results indicate that changes in gene expression induced by tretinoin could enhance survival in bladder cancer patients. Similar results were obtained using a different dataset obtained from the GEPIA2 database [27] (Figure S6).

### 3.7. Antiproliferative Effect of Tretinoin Demonstrated by C-myc Downregulation in Malignant UROtsa Cells

**Figure 7:**
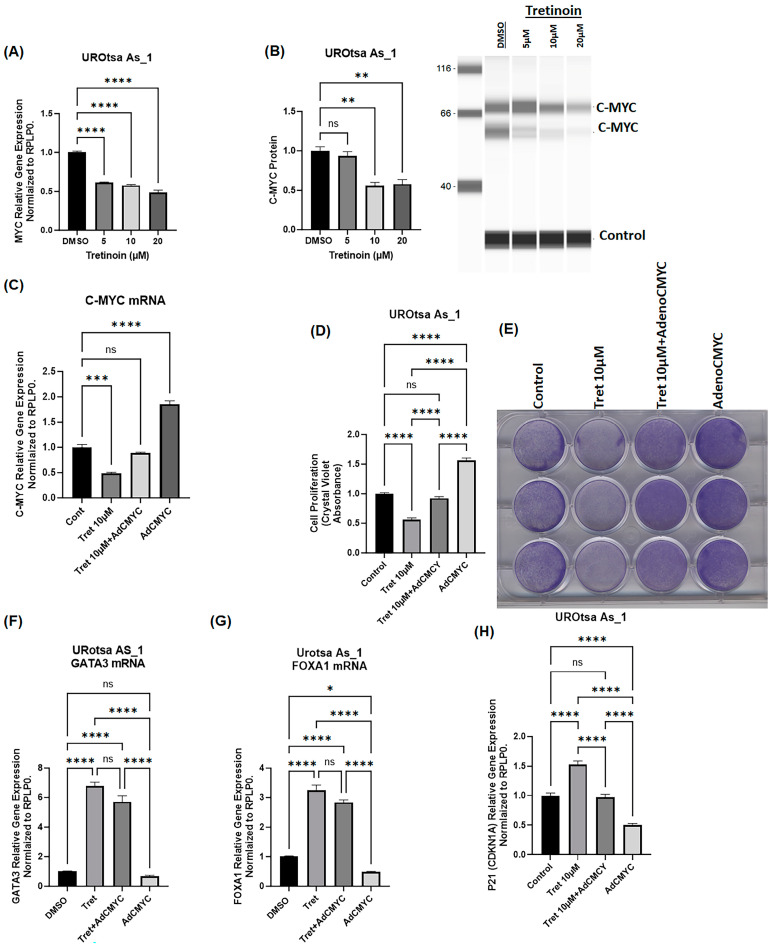
The antiproliferative effect of tretinoin is due to downregulation of c-myc in malignant UROtsa cells. (**A**) mRNA level analysis using RT-qPCR and (**B**) protein level analysis through Simple Western™ blotting of c-myc in UROtsa As_1 cells 72 h after tretinoin treatment demonstrated that tretinoin significantly reduced c-myc mRNA and protein levels (both protein bands were quantified for the purpose of c-myc protein analysis). (**C**) mRNA level analysis using RT-qPCR of UROtsa As_1 cells showing co-expression of c-myc using an adenoviral vector (AdCMYC) successfully restored c-myc mRNA levels in the presence of 10 µM of tretinoin. (**D**) Cell proliferation analysis through crystal violet staining illustrates that overexpression of c-myc using an adenoviral vector (AdCMYC) blocks the antiproliferative effect of tretinoin. (**E**) Representative micrograph of crystal violet labeling. (**F**,**G**) mRNA level analysis through RT-qPCR of *GATA3* and *FOXA1* showing that *c-myc* overexpression did not abolish the tretinoin-induced upregulation of GATA3 and FOXA1. (**H**) mRNA level analysis through RT-qPCR of the *CDKN1A* (p21) antiproliferative gene showing that c-myc overexpression abolished the tretinoin-induced upregulation of *CDKN1A* (*n* = 3, one-way ANOVA; ns = non-significant; * *p* < 0.05, ** *p* < 0.01, *** *p* < 0.001, **** *p* < 0.0001). Data represented as mean ± SEM.

C-myc is a key transcription factor that controls cell proliferation in multiple cancer cells [30], including bladder cancer cells [31]. Tretinoin has been reported to inhibit cancer cell proliferation through the myc pathway in lung cancer [32,33]. Therefore, we aimed to determine whether the effect of tretinoin on malignant UROtsa cells is due to modulation of c-myc expression. For further characterization in this experiment, we selected UROtsa As_1 because this cell isolate exhibited a clear dose-dependent response to tretinoin, and we chose a dose of 10 µM of tretinoin because this dose demonstrated a consistent effect in all of the three cell isolates. Treatment of UROtsa As_1 with 10 µM of tretinoin for 72 h significantly reduced c-myc expression at both mRNA (Figure 7A) and protein levels (Figure 7B). Interestingly, overexpression of c-myc using an adenoviral vector (Figure 7C) blocked the antiproliferative effect of tretinoin (Figure 7D). However, c-myc overexpression did not block the effect of tretinoin on the expression of *GATA3* or *FOXA1* (Figure 7F,G), indicating that c-myc downregulation was mechanistically responsible for the antiproliferative effect of tretinoin. However, the reduction in *GATA3* and *FOXA1* expression by tretinoin was independent of the c-myc axis.

## 4. Discussion

Radical cystectomy combined with neoadjuvant/adjuvant chemotherapy remains the main modality of treatment in patients with muscle-invasive bladder cancer (MIBC) [34,35]. Previously, we established a cell line model of basal muscle-invasive bladder cancer by malignantly transforming immortalized urothelial cells (UROtsa) [9]. In this study, we identified that retinoic acid (tretinoin) can reduce aggressiveness and induce luminal differentiation in malignant UROtsa cells.

Basal MIBCs are characterized by high expression of basal markers, including KRT1, KRT5, KRT6, and EGFR [2,4,35,36]. In contrast, the luminal subtype is less aggressive and characterized by the expression of luminal markers, including GATA3 and FOXA1 [2,3,4,5]. Although both subtypes are muscle-invasive, they display differences in aggressiveness, survival, and response to different modalities of neoadjuvant therapy [2,4,35].

Differentiation therapy is a modality of cancer treatment that alters the undifferentiated aggressive tumors towards a more differentiated, less aggressive phenotype that responds better to therapy. The main success has been using retinoids as differentiation therapy in acute promyelocytic leukemia [37]. The role of retinoic acid in bladder cancers remains under investigation. In vitro studies show variable effects of retinoids in different bladder cancer cells [19,21,22], with no studies dedicated to the effects of retinoids on a specific molecular subtype of bladder cancer, including basal muscle-invasive bladder cancer. The effect of retinoids needs to be investigated in each subtype of bladder cancer to determine if there is an avenue for precision therapy in bladder cancer. It has been reported that human urothelial cells (UROtsa) that are malignantly transformed through chronic arsenite exposure (UROtsa As) behave as a basal muscle-invasive bladder cancer with high expression of basal markers (KRT1, KRT5, KRT6, and EGFR) and low expression of luminal markers (GATA3 and FOXA1) [11,23,24]. Therefore, this cell line can serve as a valid cell model for aggressive basal MIBC.

Our results show that tretinoin reduces cell proliferation in arsenite-transformed malignant UROtsa cells. Other studies have demonstrated the cytostatic and antiproliferative effect of retinoids in multiple cancer cells; however, the effect was variable in bladder cancer cells. Unexpectedly, tretinoin was shown to induce cell proliferation in T24 cells [19], while it reduced proliferation in RT112 and HT-1376 cells [20,38], suggesting a cell/subtype-specific effect. In our cell model, which mimics basal MIBC, tretinoin induced a dose-dependent antiproliferative effect, mirroring the observed antiproliferative effect in RT112 and HT-1376 bladder cancer cells in other studies [20,38]. Tretinoin has been reported to induce apoptosis in T24 bladder cancer cells [21]; however, our data showed that short-term treatment with tretinoin did not induce extensive apoptosis in malignant UROtsa cells, with only one cell isolate showing a 0.5% increase in apoptosis, which we considered marginal. Whether there is an extensive apoptotic effect of tretinoin with long-term treatment will need to be explored in the future. Our data not only demonstrated the antiproliferative effect of tretinoin in malignant UROtsa cells but also identified the mechanism of the tretinoin-induced antiproliferative effect in malignant UROtsa cells, which was achieved through c-myc downregulation. Co-expression of c-myc blocked the antiproliferative effect of tretinoin in malignant UROtsa cells. Our data are consistent with the previously reported effect of retinoic acid on myc expression in other cancers [32]. C-myc has been shown to play a role in bladder cancer stem cells and aggressiveness [31], and our data extend c-myc’s role and establish its response to retinoids in basal MIBC cells.

The gene expression changes in malignant UROtsa cells after treatment with tretinoin showed a reduction in the expression of basal molecular markers (KRT1, KRT5, KRT6, EGFR) and upregulation of luminal molecular markers (FOXA1, GATA3). All-trans retinoic acid has been shown to upregulate FOXA1 in murine embryonic stem cells during urothelial differentiation [39]. Additionally, muscle-invasive and high-grade bladder cancers are characterized by a considerable reduction in FOXA1 expression compared to non-muscle-invasive bladder cancer [29]. Interestingly, the knockdown of FOXA1 in RT4 cells, a non-muscle-invasive cell model, increases cell proliferation [29]. In contrast, overexpression of FOXA1 in the aggressive T24 cells reduced cell proliferation and increased invasion [29], with all of the above supporting the beneficial effect of tretinoin-induced expression of FOXA1 in our cells. GATA3 is another luminal marker that is expressed at low levels in basal muscle-invasive bladder cancer [5,28] and in malignant UROtsa cells [24]. Our data showed dose-dependent upregulation of FOXA1 and GATA3 in malignant UROtsa cells in response to tretinoin treatment. High expression of GATA3 correlates with luminal subtype and can predict survival and therapy response in bladder cancer [40].

A clinical study identified KRT5, KRT6, and GATA3 expression as the most effective classifier in predicting luminal versus basal subtypes of bladder cancer with an accuracy of 89% [5]. Our results showed a reduction in KRT5 and KRT6 with upregulation of GATA3 in malignant UROtsa cells after tretinoin treatment, suggesting the potential of retinoids in differentiating basal MIBCs into a more luminal phenotype. However, a more extensive study on multiple cell lines and in vivo investigation are needed. Mechanistically, our results showed that the tretinoin antiproliferative effect was achieved through c-myc downregulation. However, GATA3 and FOXA1 expressions were independent of c-myc changes, suggesting an indirect effect of tretinoin on these genes or that the effect relates to another pathway.

The current study’s lack of in vivo data represents a limitation; however, our findings lay a critical foundation for future in vivo research. This includes using animal bladder cancer xenografts for the investigation of tretinoin, both as a monotherapy and in combination with established chemotherapeutics like cisplatin, which are commonly used for treating muscle-invasive bladder cancer. Such forthcoming in vivo investigations are pivotal for elucidating tretinoin’s impact on tumor growth and survival rates. Moreover, exploring the use of retinoids, whether as standalone agents or part of combination therapies, could open new avenues for enhancing treatment efficacy against bladder cancer. This approach not only promises to deepen our understanding of tretinoin’s biological mechanisms, but also aids in the future refining of therapeutic strategies for better clinical outcomes.

The potential of using retinoids in combination therapy for bladder cancer remains a promising avenue. In addition to retinoids, other drug combinations, such as PPARG agonists and trametinib, are under investigation. These combinations have demonstrated activation of the downstream retinol pathway [41], indicating the critical role of the retinoic acid pathway in the treatment of muscle-invasive bladder cancer. This evidence highlights the pathway’s potential as a therapeutic target, offering new avenues for treatment strategies.

Finally, the favorable survival analysis in patients with bladder cancer based on the gene expression profile that was extrapolated from tretinoin-treated malignant UROtsa cells supports investigation of the clinical use of tretinoin in bladder cancer.

## 5. Conclusions

Retinoids, with their role in cell growth and differentiation, present a promising potential approach to bladder cancer treatment. While challenges exist, ongoing research into combination therapies paves the way toward more effective and tolerable treatment options [18,41]. This work shows the promise of using retinoids in aggressive basal muscle-invasive bladder cancer and extends our understanding of the complex interactions between retinoids and cancer cells, which can be combined with the availability of previous data showing distorted retinol signaling in bladder cancer [16,42]. We hope this will lead to new and improved strategies in the fight against bladder cancer and encourage us to characterize the effects of different retinoids on each subtype of bladder cancer.

## Data Availability

Data are contained within the article and Supplementary Materials.

## Supplementary Materials

The following supporting information can be downloaded at: https://www.mdpi.com/article/10.3390/cancers16061178/s1; Figure S1: Effect of tretinoin treatment on UROtsa As_1 cell viability as assessed by crystal violet labeling after 24 h, 48 h, and 72 h of treatment; Figure S2: Apoptosis assay through flow cytometry analysis of annexin V and propidium iodide in UROtsa As_2 cells; Figure S3: Uncropped Simple Western™ blots that are shown in Figure 4; Figure S4: Uncropped Simple Western™ blots that are shown in Figure 5; Figure S5: Effect of tretinoin in UROtsa As_2 cells; Figure S6: Kaplan–Meier survival analysis using GEPIA2; Table S1: Primers used in gene expression analysis; Table S2: Antibodies used in Simple Western™ blotting.
