# Supplementary material for: The Effect of Retinoic Acid on Arsenite-Transformed Malignant UROtsa Bladder Cancer Cells: In Vitro Model of Basal Muscle-Invasive Bladder Cancer
Source: Cancers (Basel). 2024 Mar 17;16(6):1178. doi: 10.3390/cancers16061178 (PMC10969336; doi:10.3390/cancers16061178)
Supplement: Supplementary file 1 [file cancers-16-01178-s001.zip › cancers-2896974-supplementary.pdf]

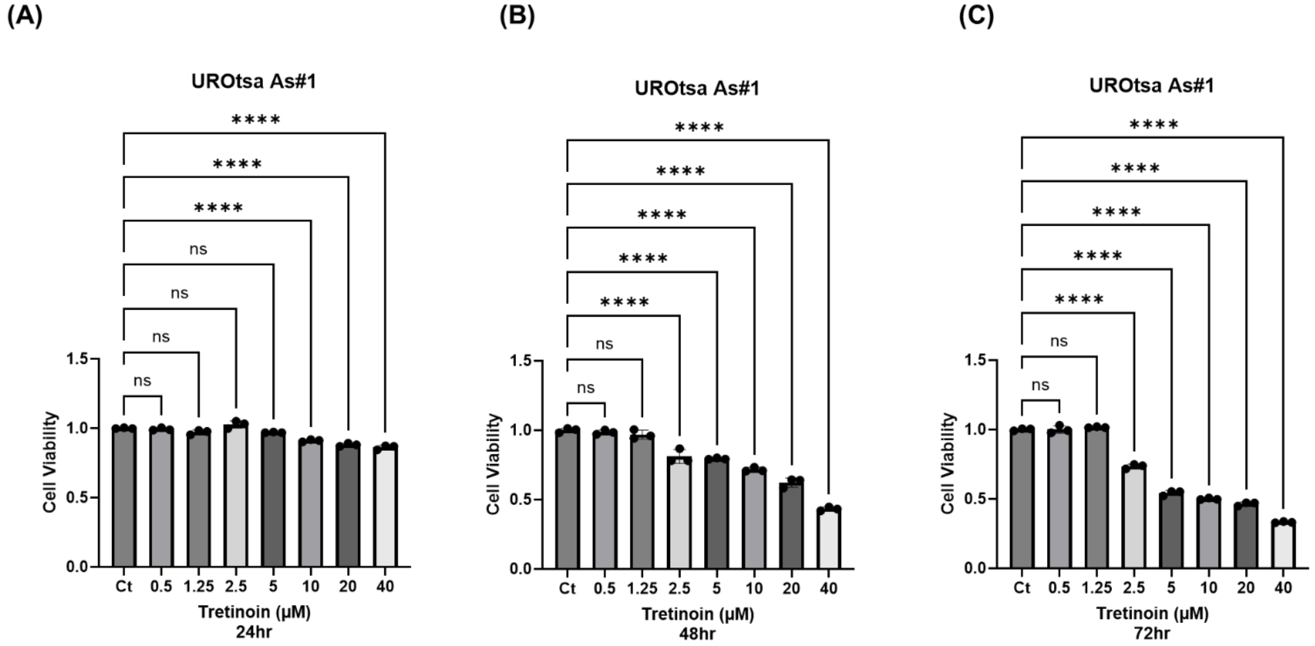

Figure S1: Effect of tretinoin treatment on cell proliferation of UROtsa As_1 cells, assessed by crystal violet labeling after treatment with tretinoin for **(A)** 24 hours, **(B)** 48 hours, and **(C)** 72 hours. Tretinoin was applied at 0.5, 1.25, 2.5, 10, 20, and 40 μM, and cell proliferation was quantified by measuring the absorbance of crystal violet at 570 nm. One-way ANOVA was used to compare treated groups to the DMSO control at each time point (ns = not significant; ****$p < 0.0001$).

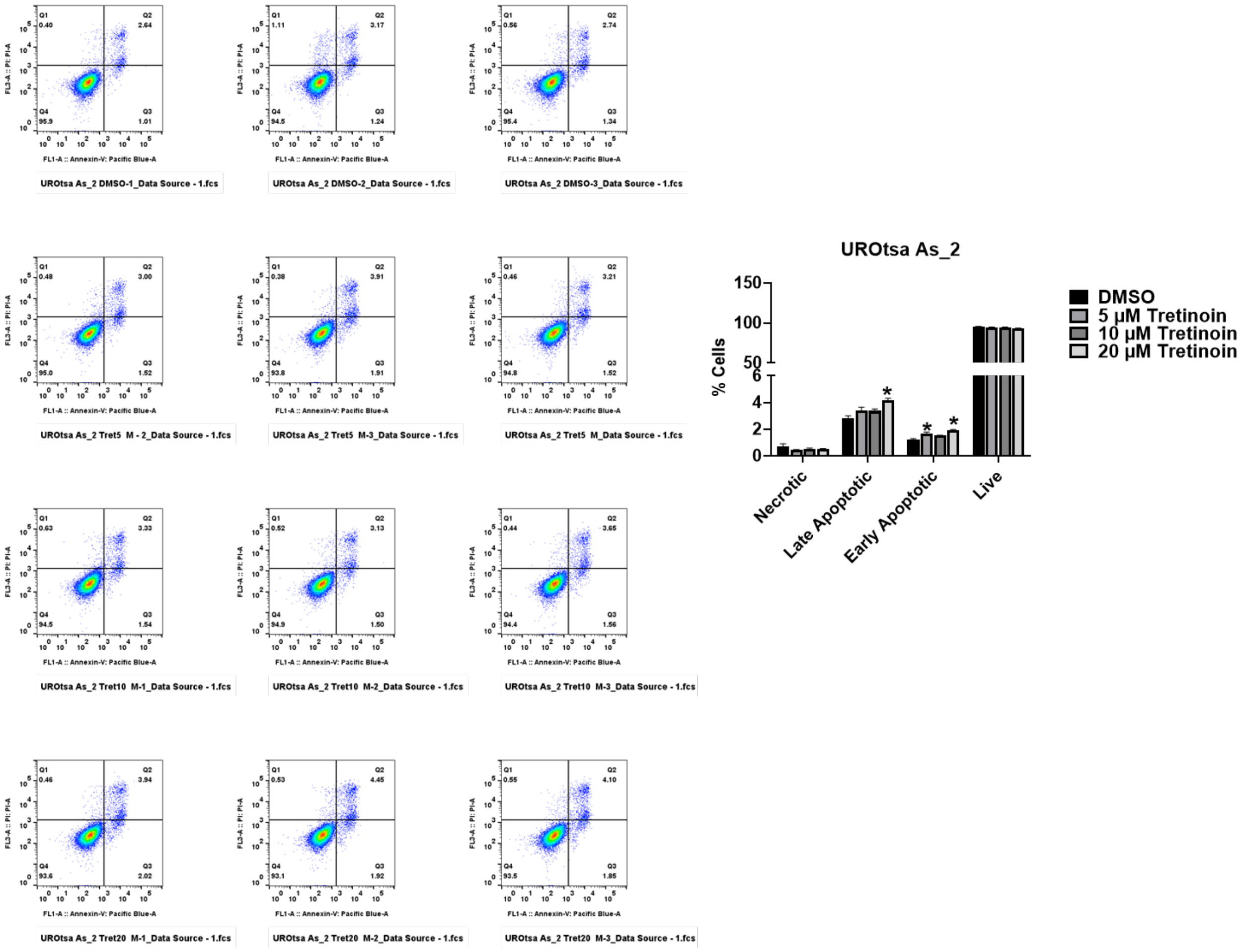

Figure S2: Figure S2: Apoptosis assay via flow cytometry analysis assessing annexin V and propidium iodide staining in UROtsa As_2 cells after treatment with 5, 10, and 20 µM tretinoin for 48 hours. Results indicate that tretinoin induces apoptosis in UROtsa As_2 cells in a minimal but statistically significant manner. Data are represented as mean ± SEM (n = 3). Statistical significance was assessed using one-way ANOVA (*p < 0.05).

(4E)

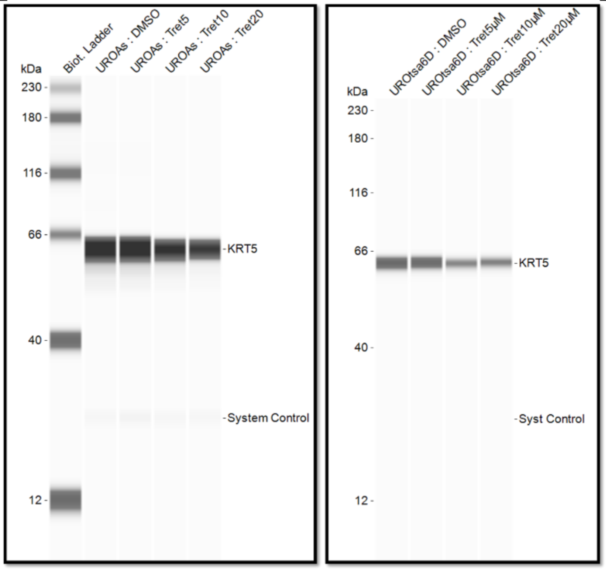

(4F)

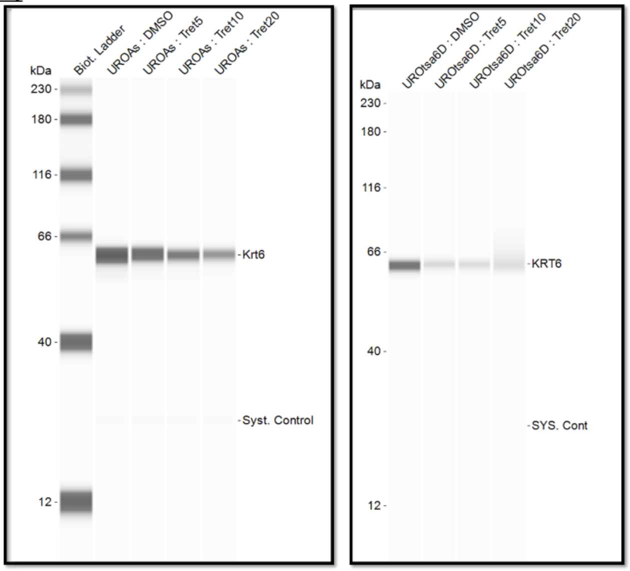

(4G)

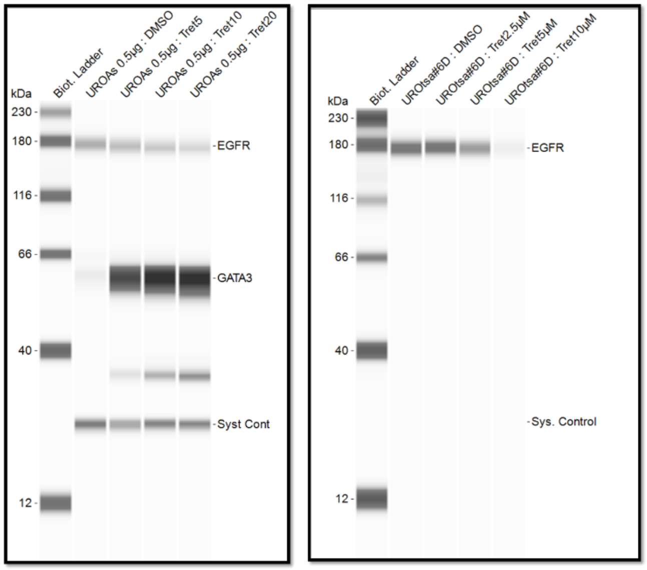

<u>Figure S3</u>: Uncropped blots Simple Western™ that are shown in figure 4.

(5C)

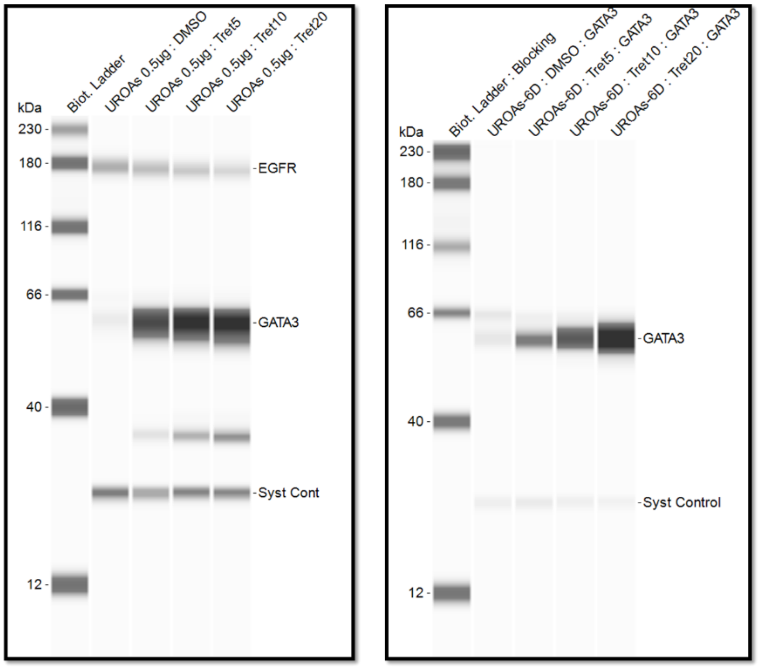

(5D)

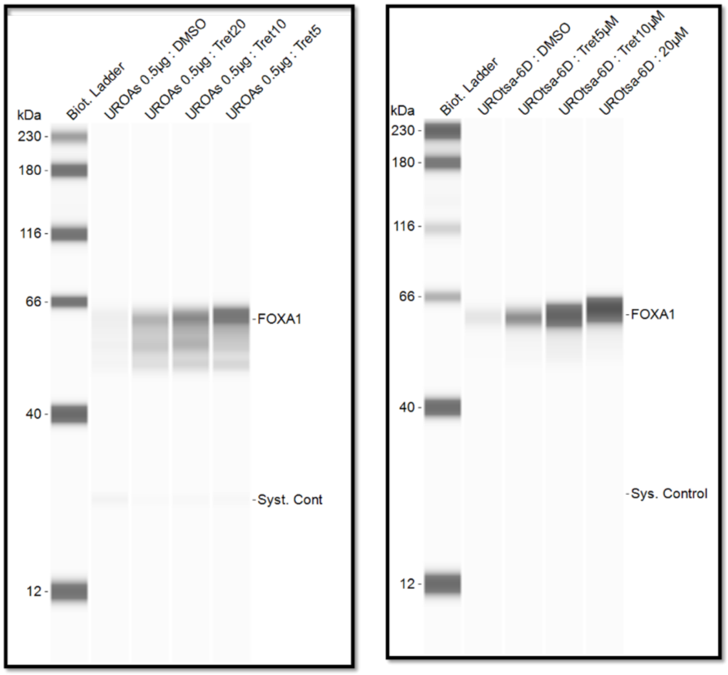

<u>Figure S4:</u> Uncropped blots of Simple Western™ that are shown in figure 5.

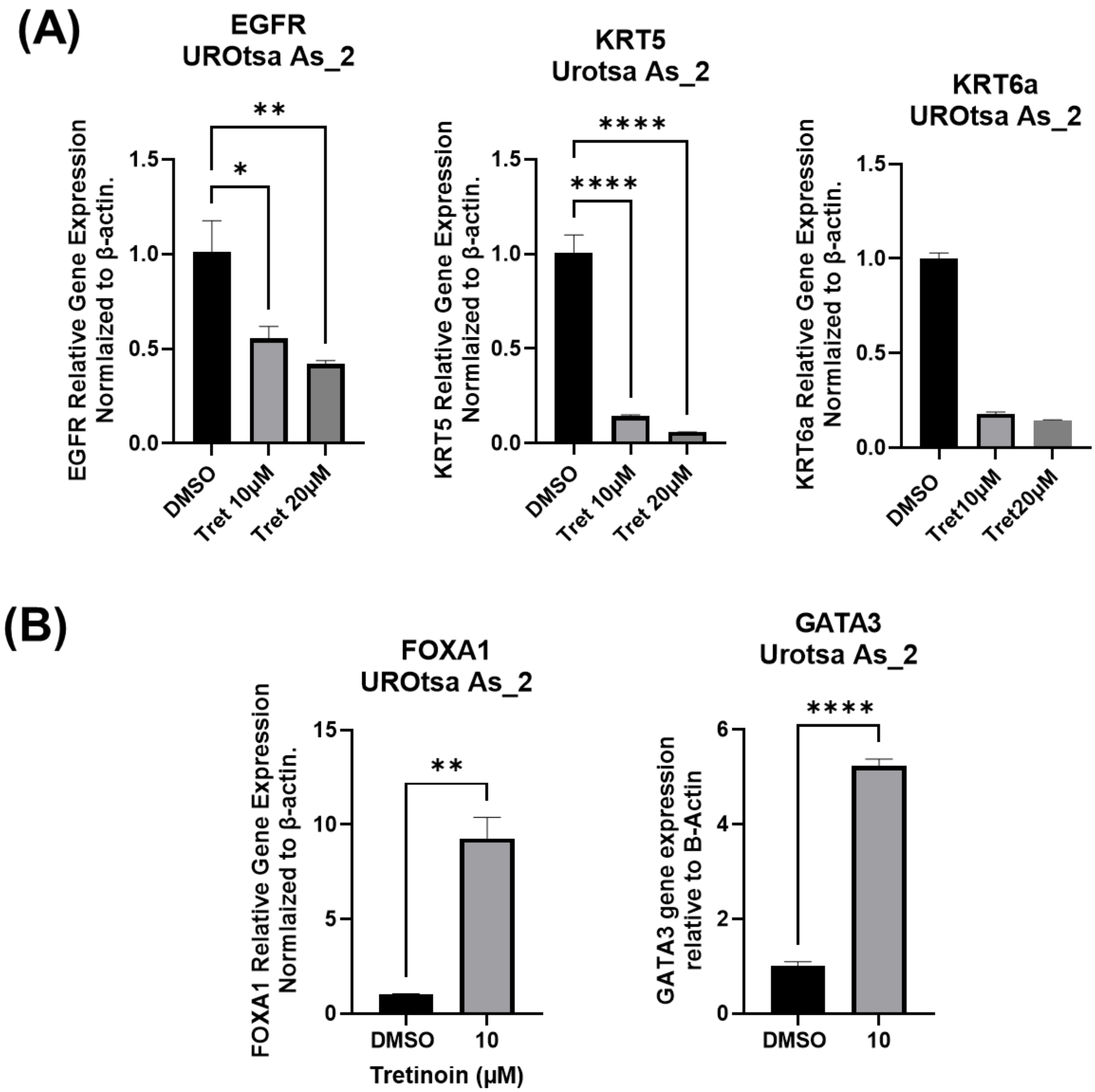
(A)
EGFR
UROtsa As_2
EGFR Relative Gene Expression
Normlaized to β-actin.
1.5
1.0
0.5
0.0
**
*
DMSO
Tret 10µM
Tret 20µM
KRT5
Urotsa As_2
KRT5 Relative Gene Expression
Normlaized to β-actin.
1.5
1.0
0.5
0.0
****
****
DMSO
Tret 10µM
Tret 20µM
KRT6a
UROtsa As_2
KRT6a Relative Gene Expression
Normlaized to β-actin.
1.5
1.0
0.5
0.0
DMSO
Tret10µM
Tret20µM
(B)
FOXA1
UROtsa As_2
FOXA1 Relative Gene Expression
Normlaized to β-actin.
15
10
5
0
**
DMSO
10
Tretinoin (µM)
GATA3
Urotsa As_2
GATA3 gene expression
relative to B-Actin
6
4
2
0
****
DMSO
10

(C)

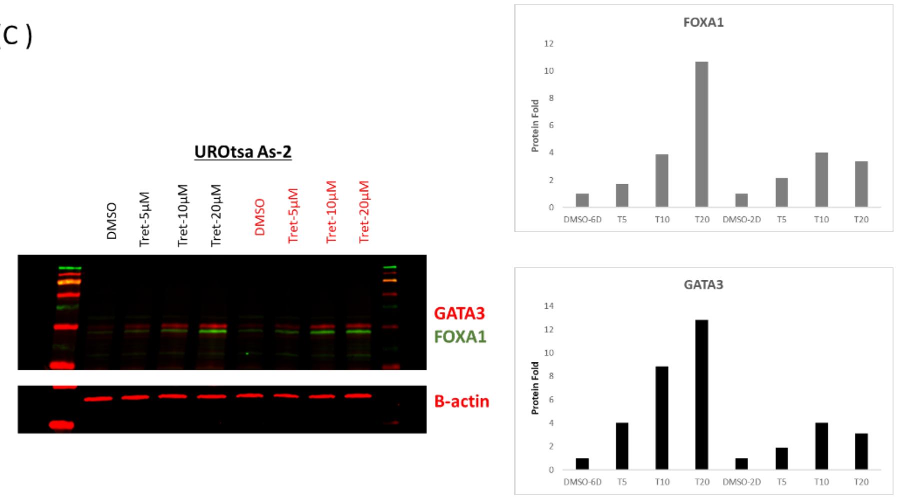

Figure S5: effect of tretinoin in UROtsa As_2 cells.

mRNA levels analysis by RT-QPCR in UROtsa As_2 treated with tretinoin in indicated does for 48 hours of **(A)** EGFR, KRT5, KRT6a **(B)** FOXA1 and GATA3 after treatment with 10 µM tretinoin. **(C)** Protein analysis by western blot of GATA3 and FOXA1 in UROtsa As_2, 48- hours after treatment with DMSO (DMSO) as control or tretinoin (5, 10, 20 µM).

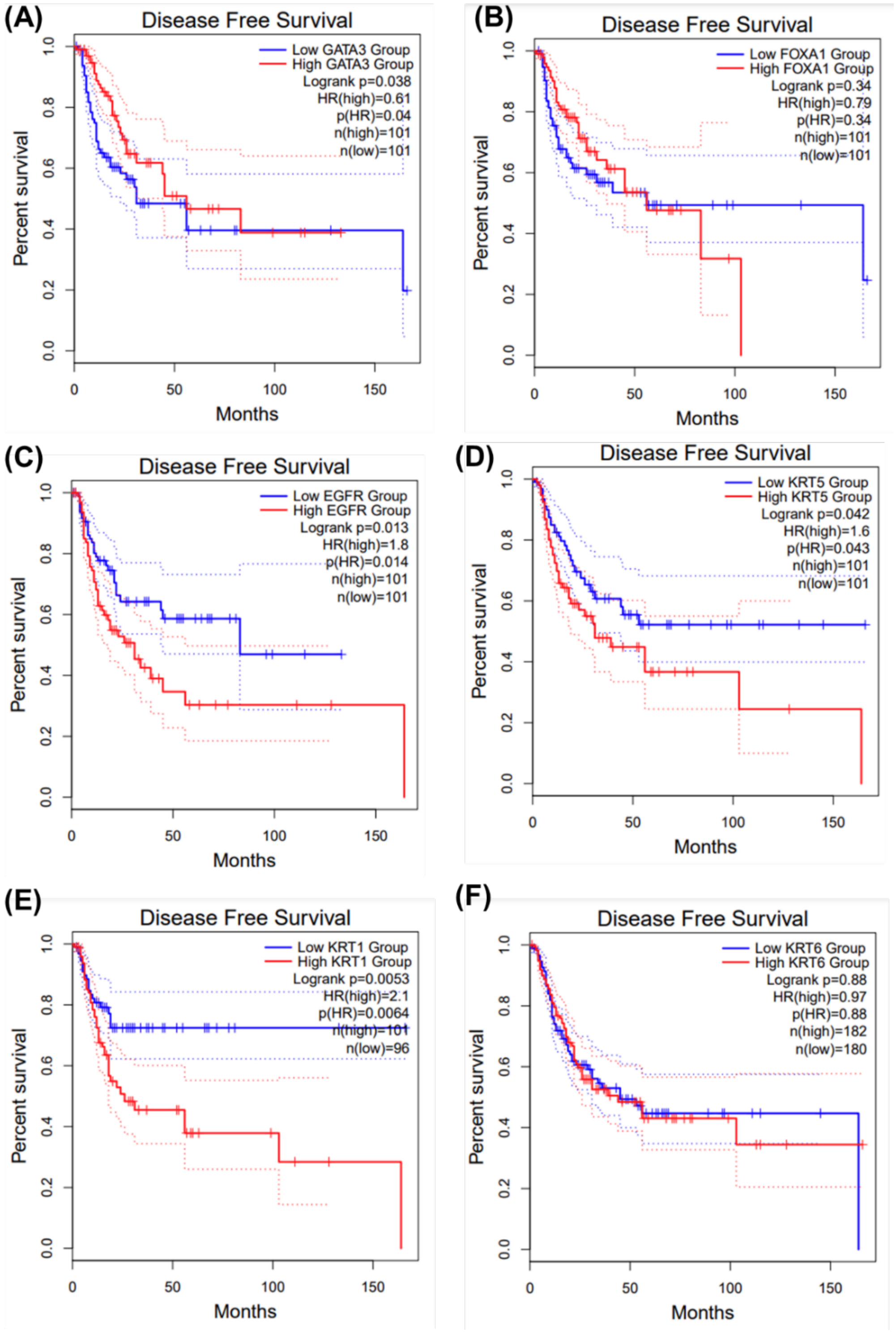

Figure S6: Kaplan-Meier Survival analysis using GEPIA2 (http://gepia2.cancer-pku.cn/#survival) database showing the correlation of the expression of (A) GATA3, (B) FOXA1, (C) EGFR, (D) KRT5, (E) KRT1, and (F) KRT6 with survival in patients with bladder cancers.

Table S1: Primers used in gene expression analysis.

Gene	Catalog No./unique Assay ID	Source
KRT5	Hs.PT.58.14446018	Integrated DNA Technologies
KRT6a	Hs.PT.58.26132549	Integrated DNA Technologies
KRT1	Hs.PT.58.24741966	Integrated DNA Technologies
EGFR	Hs.PT.58.15419889	Integrated DNA Technologies
FOXA1	Hs.PT.58.1788586	Integrated DNA Technologies
GATA3	Hs.PT.58.4308511	Integrated DNA Technologies
CMYC	Hs.PT.58.26770695	Integrated DNA Technologies
CDKN1A	Hs.PT.58.40874346.	Integrated DNA Technologies
RPLP0	Hs.PT.39a.22214824	Integrated DNA Technologies
ACTB	qHsaCED0036269	BIO-RAD

Table S2: Antibodies used in Simple Western™.

Protein	Antibody	Dilution	Protein loading Conc
KRT5	Invitrogen PA5-29670	1:1600	0.05µg/µl.
KRT6	Sanat Cruz Cat#sc-514520	1:1600	0.05µg/µl.
EGFR	Cell Signaling Cat#4267S	1:300	0.5µg/µl.
FOXA1	Santa Cruz Cat#SC-101058	1:50	0.5µg/µl.
GATA3	Cell Signaling Cat#5852	1:50	0.5µg/µl.
